# AoUPRS: A cost-effective and versatile PRS calculator for the *All of Us* Program

**DOI:** 10.1186/s12864-025-11693-9

**Published:** 2025-05-22

**Authors:** Ahmed Khattab, Shang-Fu Chen, Nathan Wineinger, Ali Torkamani

**Affiliations:** 1 Integrative Structural and Computational Biology, Scripps Research, La Jolla, CA USA; 2 Scripps Research Translational Institute, 3344 North Torrey Pines Court, Suite 300, La Jolla, CA 92037 USA

**Keywords:** Polygenic Risk Score (PRS), All of Us (AoU) Program, Cost-Effective Genomics, Scalable PRS Calculation

## Abstract

**Background:**

The *All of Us* (AoU) Research Program provides a comprehensive genomic dataset to accelerate health research and medical breakthroughs. Despite its potential, researchers face significant challenges, including high costs and inefficiencies associated with data extraction and analysis. AoUPRS addresses these challenges by offering a versatile and cost-effective tool for calculating polygenic risk scores (PRS), enabling both experienced and novice researchers to leverage the AoU dataset for large-scale genomic discoveries.

**Methods:**

We evaluated three PRS models from the PGS Catalog (coronary artery disease, atrial fibrillation, and type 2 diabetes) using two distinct approaches in the Hail framework: MatrixTable (MT), a dense representation, and Variant Dataset (VDS), a sparse representation optimized for large-scale genomic data. Computational cost, resource usage, and processing time were compared. To assess the similarity of PRS performance between these two approaches, we compared odds ratios (ORs) and area under the curve (AUC). Lin’s concordance correlation coefficient (CCC) was also computed to quantify agreement between PRS scores generated by MT and VDS.

**Results:**

The VDS approach reduced computational costs by up to 99.51% (e.g., from $32 to $0.036 for a 51-SNP score) while maintaining PRS estimates that were highly similar to those obtained using the MT approach. Across all three PRS models, AUC comparisons showed minimal differences between MT and VDS, indicating that both approaches yield consistent PRS performance. Agreement between PRS scores calculated by both approaches was further supported by Lin’s CCC values ranging from 0.9199 to 0.9944, confirming strong concordance. Empirical cumulative distribution function (ECDF) plots further illustrated the near-identical distribution of PRS values across methods.

**Conclusions:**

AoUPRS enables efficient and cost-effective PRS computation within AoU, providing substantial cost savings while maintaining highly consistent PRS estimates. These findings support the use of AoUPRS for large-scale genomic risk assessment, making the AoU dataset more accessible and practical for diverse research applications. The tool’s open-source availability on GitHub, coupled with detailed documentation and tutorials, ensures accessibility and ease of use for the scientific community.

**Supplementary Information:**

The online version contains supplementary material available at 10.1186/s12864-025-11693-9.

## Introduction

The *All of Us* (AoU) Research Program [1, 2], initiated by the National Institutes of Health (NIH), aims to accelerate health research and medical breakthroughs by creating a comprehensive phenotypic and genomic dataset, broadly accessible to researchers and the public. This resource includes over 245,000 short-read whole genome sequencing (srWGS) samples available in formats such as Variant Call Format (VCF), Hail MatrixTable (MT), and PLINK—all hosted on the Google Cloud Platform (GCP).

Polygenic risk score (PRS) analysis has been extensively conducted in other large, accessible databases such as the UK Biobank [3], demonstrating the clinical validity of PRSs [4]. However, genomic researchers face significant challenges when executing PRS calculations with AoU data. All work must be conducted on the AoU Workbench, and extracting and downloading samples to local workbenches incurs high costs. Existing tools struggle with the dataset’s scale, leading to inefficiencies and additional expenses. This contrasts with the ease and low cost of accessing health data, surveys, and other phenotypic datasets through the user-friendly AoU workbench. Consequently, there is a significant barrier, both in complexity and costs, to executing PRS calculations on AoU data.

To address these challenges, we developed AoUPRS, a versatile and cost-effective PRS calculator tool tailored for the AoU dataset. This tool is designed to facilitate both experienced and novice researchers in leveraging WGS data in AoU for genomic discoveries. Here, we present the development, implementation, and evaluation of AoUPRS, highlighting its versatility, cost-effectiveness, and potential impact on genomic research.

## Methods

### Overview of hail Matrixtable (MT) and Variant Dataset (VDS) formats

Hail [5], a scalable framework for genomic data analysis, supports two primary data formats: MatrixTable (MT) and Variant Dataset (VDS). The MT format is a dense representation where each cell (sample–variant pair) contains genotype information. This format is ideal for datasets with high variant density and allows efficient querying and manipulation of genetic data. However, it can be computationally expensive and less efficient for large-scale datasets. Accessing Hail data in this format is currently presented in AoU tutorials for beginning researchers.

In contrast, the VDS format is a sparse representation optimized for storing large genomic datasets with many variants. It uses a more compact structure where only non-reference calls are stored, significantly reducing storage requirements and computational costs. This makes the VDS format more suitable for large-scale studies. However, because the MT in AoU is populated from VDS with a minimum allele frequency and count threshold, VDS better preserves rare and ultra-rare variants, while MT is optimized for more common variants.

All genomic data used in this study underwent pre-processing and quality control (QC) as implemented by the AoU Research Program. We excluded all samples flagged by the AoU team for quality concerns, following the guidelines outlined in the *AoU Genomic Quality Report* [6], which documents the QC pipeline used to generate and curate the dataset. Additionally, while the MT format contains only variants that passed AoU’s QC filtering, the VDS includes all variants, regardless of their QC status. The filtered MT dataset contained 48,314,438 high-confidence variants, consistent with AoU documentation [7], while the unfiltered VDS contained over 1 billion variant sites (1,031,611,675).

To ensure that only QC-passed variants were used in downstream calculations, we queried the Variant Annotation Table (VAT) [8], which includes only passed variants, for those present in the corresponding PRS weight table. This approach effectively served as an indirect filter on the VDS, allowing us to isolate high-confidence variants for each score without processing or densifying the entire dataset.

After all filtering steps, the final dataset included 193,835 short-read WGS (srWGS) samples linked to electronic health record (EHR) data. To optimize computational efficiency, a subset of 1,000 samples was randomly selected for evaluating smaller PRS scores.

### AoUPRS: Approaches for PRS calculation

AoUPRS provides two distinct approaches for calculating PRS using the AoU dataset. Each approach leverages the strengths of the respective Hail data formats to balance computational efficiency and cost-effectiveness. In both approaches, effect allele counts are multiplied by their corresponding weights to compute weighted counts. The total PRS for each sample is then calculated by summing the weighted counts across all relevant variants. The results—including total PRS and the number of variants used—are written to an output file with an option to export all found variants contributing to the PRS for further analysis.

### Approach 1: Using hail dense Matrixtable (MT)

In the first approach, PRS weights are imported as a Hail Table and annotated with variant information, including effect alleles and their weights. The MT is filtered to retain only the variants present in the PRS weights table. The tool then calculates the effect allele count for each variant by comparing the reference and alternate alleles against the PRS weight table, handling different genotypic scenarios—such as homozygous reference, homozygous alternate, and heterozygous genotypes.

### Approach 2: Using hail sparse Variant Dataset (VDS)

In the second approach, the VAT provided by the AoU Research Program is utilized. The VAT contains comprehensive annotations for all variants in the dataset, including variant identifiers, allele frequencies, and annotations across different population subgroups. Since the VDS contains all variants, including those that did not pass quality control, we use the VAT to filter for high-quality variants, ensuring high-quality PRS calculations while optimizing efficiency. This filtering step removes low-confidence variants that failed AoU’s QC filtering, preventing their inclusion in downstream analyses.

PRS weights are imported as a Hail Table, and the VDS is filtered based on the loci specified in the PRS weights table using interval queries. The tool handles missing genotype calls by assuming they represent homozygous reference genotypes and calculates effect allele counts similarly to the MT approach.

### Statistical analysis

To assess the association between PRS and disease outcomes, we performed logistic regression analysis, reporting odds ratios (ORs) with 95% confidence intervals (CIs). No additional covariates were included in the models; PRS was the sole predictor variable used to evaluate association and predictive performance. To compare PRS performance between the MT and VDS approaches, we used the area under the curve (AUC) from receiver operating characteristic (ROC) analysis. To evaluate agreement between PRS scores generated by MT and VDS, we computed Lin’s concordance correlation coefficient (CCC), which quantifies both precision and accuracy. A CCC value close to 1 indicates near-perfect concordance.

Additionally, we examined empirical cumulative distribution function (ECDF) plots to visually compare PRS distributions across methods. ECDF plots illustrate the proportion of individuals below each PRS value, allowing for direct comparisons of distributional shifts. While CCC provides a numerical measure of agreement, ECDF plots offer insights into potential systematic differences in PRS distributions.

All statistical analyses were conducted in Python 3.10 using the following packages: scikit-learn for computing AUC (roc_auc_score), statsmodels for logistic regression, and scipy for Pearson correlation (pearsonr), which was used in the calculation of CCC.

## Results

### Cost and performance

To our knowledge, no previously published studies have provided explicit cost estimates for PRS calculations within the AoU Research Program. To address this gap, our study offers an assessment of computational expenses, demonstrating that AoUPRS achieves substantial cost savings. To evaluate its performance and cost-effectiveness, we compared three different PRS scores using both the Hail MT and VDS approaches, assessing cost, computational resources, and processing time. The results are summarized in Table 1.

The results demonstrate that the VDS approach is significantly more cost-effective compared to the MT approach. For instance, the VDS approach achieved a cost reduction of 99.51% for a 51-SNP score, reducing costs from approximately $32 to $0.036. Similarly, for a score comprising 1,108,235 SNPs, the cost dropped by 85%, from $50 to $7.50. The VDS format’s sparse representation minimizes computational costs, making it more suitable for large-scale studies. However, it is important to note that while the VDS approach is much cheaper, filtering intervals in the VDS tends to be slower with large weight tables, which increases the time required for calculations compared to the MT approach.

### Performance evaluation and predictive power

We assessed the performance of AoUPRS by evaluating the association between PRS and disease outcomes for three scores from the PGS Catalog [9]: PGS000746 [10], PGS002774 [11], and PGS004859 [12] corresponding to coronary artery disease (CAD), atrial fibrillation (AF), and type 2 diabetes (T2D), respectively. 

Logistic regression analysis was conducted using PRS scores generated from both the Hail MT and VDS datasets. For PGS000746, which is associated with CAD, the OR was 1.090 with a 95% CI of [1.077, 1.104] using MT, and 1.092 with a 95% CI of [1.079, 1.106] using VDS. For PGS002774, associated with AF, the OR was 1.794 with a 95% CI of [1.712, 1.879] using MT, and 1.785 with a 95% CI of [1.704, 1.870] using VDS. For PGS004859, associated with T2D, both approaches yielded an OR of 1.096 with a 95% CI of [1.072, 1.120]. These results indicate that both methods yielded similar predictive power and significance levels.

**Table 1:** Cost, resources, and time for different scores using hail MT and VDS approaches

Score	Score Size (SNPs)	Sample Size	Approach	Main Node^a^	Cluster Resources^b^	Time (min)	Cost ($)^c^	Cost Reduction (%)^d^
PGS004226	51	1,000	MT	4 CPUs, 15 GB	200 / 0	40	32	
			**VDS**		**2 / 50**	**3**	**0.036**	99.51
PGS000746	1,938	193,835	MT	4 CPUs, 26 GB	300 / 0	34	41.3	
			**VDS**		**2 / 50**	**4**	**0.35**	99.15
PGS002774	216,487	193,835	MT	4 CPUs, 26 GB	300 / 0	34	41.3	
			**VDS**		**2 / 50**	**20**	**1.76**	95.75
PGS004859	1,108,235	193,835	MT	4 CPUs, 15 GB	300 / 0	38	50	
			**VDS**	4 CPUs, 26 GB	**2 / 50**	**76**	**7.5**	85

^a^ Main Node: Number of CPUs and RAM in GB

^b^ Cluster Resources: Number of (**workers** / **preemptible workers**) nodes and their specifications (**4 CPUs** and **15 GB RAM**)

^c^ Total computational costs in U.S. dollars ($)

^d^ Cost Reduction (%): Percentage reduction in cost when using VDS compared to MT

In addition to ORs, we assessed the predictive performance of PRS using AUC. The results across all three PRS scores showed highly similar performance between the MT and VDS approaches. For PGS000746, the AUC was 0.5385 for MT and 0.5390 for VDS. For PGS002774, the AUC was 0.5667 for MT and 0.5656 for VDS. Similarly, for PGS004859, the AUC values were nearly identical at 0.5193 (MT) and 0.5195 (VDS). These findings confirm that the predictive performance of PRS is nearly indistinguishable between the MT and VDS methods, reinforcing the equivalence of both approaches in terms of model performance.

To further evaluate the agreement between PRS scores generated by the two approaches, we computed CCC. The CCC values for PGS000746, PGS002774, and PGS004859 were 0.9809, 0.9199, and 0.9944, respectively, indicating a high degree of concordance between MT and VDS PRS scores. These findings suggest that despite differences in computational efficiency, both approaches yield highly similar PRS estimates.

Additionally, we examined ECDF plots for the PRS values of the three scores plus PGS004226 [13] to visualize PRS distributions across methods. ECDF represents the proportion of observations that fall below a given value, providing a complete view of the cumulative distribution of PRS scores. While CCC provides a numerical assessment of agreement, ECDF plots offer insights into potential systematic shifts in PRS distributions. The ECDF curves for MT and VDS PRS scores were closely aligned (Supplemental Fig. 1), reinforcing the finding that both methods provide comparable results in terms of predictive power and distribution of PRS values.

## Discussion

The high concordance between PRS scores from MT and VDS, as indicated by Lin’s CCC values, suggests that both methods produce highly similar results. However, key differences exist in variant retention and computational efficiency. VDS retains all variants, including rare and ultra-rare ones, whereas MT in AoU is derived from VDS but includes an imposed Alternate Allele Count Frequency (ACAF) threshold (AF > 1%, AC > 100), making it more suited for common variant analyses.

Beyond variant representation, VDS offers substantial advantages in cost-effectiveness and scalability. Its sparse structure significantly reduces computational expenses, making it an optimal choice for large-scale or budget-limited studies. Additionally, VDS assumes all ‘no calls’ are homozygous reference, which can introduce minor discrepancies in PRS scores compared to MT due to differences in handling missing genotypes. However, the high CCC values indicate that these differences have a minimal impact on overall PRS estimation.

The choice between MT and VDS should be guided by study objectives and computational constraints. MT is beneficial for analyses requiring comprehensive genotype data and is ideal for studies with high variant density. In contrast, VDS is particularly advantageous for high-throughput studies, where efficiency and cost reduction are priorities. Additionally, VDS maximizes computational resources, making it a practical choice for pilot studies or projects with budgetary limitations as it allows researchers to maximize their $300 free computing credit without exhausting resources as quickly as with the MT approach.

Overall, our findings demonstrate that while MT and VDS differ in computational and structural aspects, they produce nearly identical PRS results. Researchers can confidently use VDS for cost-effective and scalable PRS calculations without compromising accuracy, making it a practical alternative for large-scale genomic studies.

## Conclusion

Both Hail MT and VDS are powerful tools for PRS calculation, each with unique strengths and limitations. The choice between them should be guided by the specific requirements of the study, considering factors such as variant density, computational cost, and the importance of capturing low-frequency variants. AoUPRS demonstrates both versatility and cost-effectiveness, facilitating genomic discoveries within the AoU dataset.

### Availability and requirements

#### Project name

AoUPRS

#### Project home page

https://github.com/AhmedMKhattab/AoUPRS

#### Operating system(s)

Platform independent

#### Programming Language

Python

#### Other requirements

Hail framework

#### License

MIT

## Electronic supplementary material

Below is the link to the electronic supplementary material.

Supplementary Material 1

## Data Availability

No datasets were generated or analysed during the current study.
